# Variations in SXT elements in epidemic *Vibrio cholerae* O1 El Tor strains in China

**DOI:** 10.1038/srep22733

**Published:** 2016-03-09

**Authors:** Ruibai Wang, Dong Yu, Junjie Yue, Biao Kan

**Affiliations:** 1 State Key Laboratory for Infectious Disease Prevention and Control, National Institute for Communicable Disease Control and Prevention, Chinese Centre for Disease Control and Prevention, Beijing 102206, P. R. China; 2 Beijing Institute of Biotechnology, State Key Laboratory of Pathogen and Biosecurity, Beijing 100071, P. R. China; 3 Collaborative Innovation Centre of Diagnosis and Treatment of Infectious Diseases, Hangzhou, China

## Abstract

*Vibrio cholerae* O1 El Tor biotype strains are responsible for three multiyear epidemics of cholera in China during the seventh ongoing pandemic. The presence of the integrative conjugative element SXT is strongly correlated with resistance to nalidixic acid, tetracycline, and trimethoprim-sulfamethoxazole in these strains. Here, we sequenced the conserved genes of the SXT element, including *eex*, *setR*, and *int*, from 59 *V. cholerae* O1 El Tor strains and extracted and assembled the intact SXT sequences from the 11 genome sequenced strains. These elements had characteristics distinct from those of previously reported integrative conjugative elements (ICEs). They could be clearly divided into two types based on the clustering of conserved genes and gene structures of the elements, showing their possibly independent derivation and evolution. These two types were present before and after 2005, respectively, demonstrating the type substitution that occurred in 2005. Four to six antibiotic-resistant genes were found on the SXT elements, including genes resistant to tetracycline, trimethoprim-sulfamethoxazole, and multiple drugs. In summary, our findings demonstrated the roles of the SXT element in the emergence of multidrug resistance in epidemic O1 El Tor *V. cholerae* strains in China.

Cholera is an infectious disease caused by the gram-negative pathogen *Vibrio cholerae*. Cholera infection leads to severe dehydrating diarrhea. *V. cholerae* has caused seven disastrous pandemics in recorded history and remains a major threat to public health in developing countries. Based on the somatic O antigen, *V. cholerae* strains can be classified into more than 200 serogroups, although only serogroups O1 and O139 are associated with epidemic infections. The El Tor biotype of serogroup O1 is responsible for the seventh ongoing cholera pandemic and has spread throughout China since 1961. This situation continues to worsen owing to the emergence and spread of drug-resistant *V. cholerae* strains. Unlike the serogroup O139 strains, O1 El Tor isolates from China exhibit lower antibiotic resistance, except against nalidixic acid, tetracycline, and trimethoprim-sulfamethoxazole. Interestingly, resistance to all three antibiotics has been shown to be strongly correlated with the presence of the SXT element^1^.

The SXT element (conferring resistance to sulfa and trimethoprim) is an integrative conjugative element (ICE) belonging to the SXT/R391 family. SXT (99.5 kb in length), which was identified from a *V. cholera*e O139 clinical strain, MO10, isolated in India in 1992, and R391 (89.5 kb), which was identified from *Providencia rettgeri*, were the first reported SXT/R391 family ICEs^2^,^3^. SXT has been found in most clinical and environmental isolates of *V. cholerae*, including serogroup O1 from both Asian and African isolates. SXT has also been detected in other diverse species of gammaproteobacteria, such as *Photobacterium damselae*, *Shewanella putrefaciens*, and *Providencia alcalifaciens*^4^. To date, more than 30 SXT/R391 family ICEs have been sequenced.

The SXT/R391 family is characterized by a conserved site-specific integrase that mediates integration into the 5′-end of the *prfC* gene of the host chromosome; this gene encodes peptide chain release factor 3, which is responsible for the integration and excision roles found in lambdoid phages^5^. SXT/R391 ICEs can be transferred between bacteria by conjugation, resulting in the transfer of a diverse array of functions to the host. Resistance to multiple antibiotics and heavy metals were the first described fitness functions in ICEs of this family^6^. Subsequently, functions involved in regulation of motility and biofilm formation were identified^7^.

Elements of the SXT/R391 family share a genetic backbone of 52 syntenic genes, which mediate their integration/excision, conjugative transfer, and regulation during the life cycle of the ICEs^2^. In addition to this conserved core scaffold, these elements also harbor variable fragments, such as antibiotic resistance genes. The variable regions range from 30 to 60 kb in length and are mostly found at five insertion hotspots (HSs), termed HS1, HS2, HS3, HS4, and HS5, and four variable regions, named VRI, VRII, VRIII, and VRIV^8^.

Determination of the sequences of the ICE of the SXT/R391 family is essential for elucidating their organization and evolution. Though, an elaborate ICE capture system on plasmids has been developed to facilitate their sequencing and has been used for determining five ICE sequences^8^, it is an arduous task due to their size and predominantly chromosomal localization. In this study, we explored the types and evolution of SXT elements in Chinese *V. cholerae* O1 strains by clustering the backbone genes of the SXT element. We also extracted 11 complete SXT sequences from a genomic resequencing database of *V. cholerae* O1 El Tor strains established in our laboratory^9^. Our SXT genomic structure analysis described the differences between two sequence types of SXT elements, positioned the antibiotic resistance genes on the SXT elements, and presented the key role of this ICE in the emergence and transmission of multidrug resistance in *V. cholerae*.

## Results and Discussion

### Clustering of SXT elements by three conserved genes

**Figure 1:**
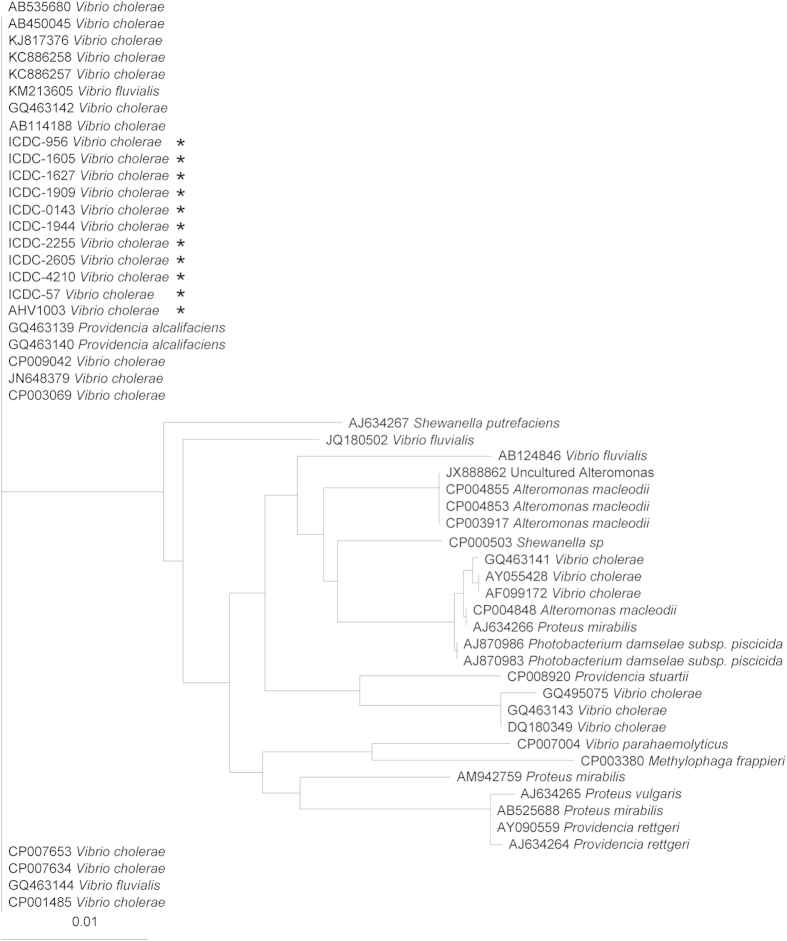
Clustering tree of the integrase gene (*int*) of SXT elements, including the 11 genomic SXT elements identified in this study and 43 ICEs in the GenBank database with 1242-bp full-length *int* sequences.

The *int* gene encodes the integrase enzyme of the SXT element. This enzyme is required for the site-specific integration of the SXT element into the host chromosome and its excision from the chromosome^10^. Using primers designed for the *int* gene, the complete CDS regions of the *int* genes of the tested strains were amplified and sequenced. Alignment results showed that all of the Chinese strains had completely identical *int* gene sequences without any mutations in nucleotides. We then compared the *int* gene of our isolates with 43 full-length *int* gene sequences (1242 bp) in the GenBank database (Fig. 1) and found that the SXT elements of the Chinese strains were grouped together in a cluster primarily composed of SXT elements from *V. cholerae*. The SXT elements could be separated from those identified in *Alteromonas macleodii*, *Shewanella*, and *Providencia*, but not in *P. alcalifaciens*.

**Figure 2:**
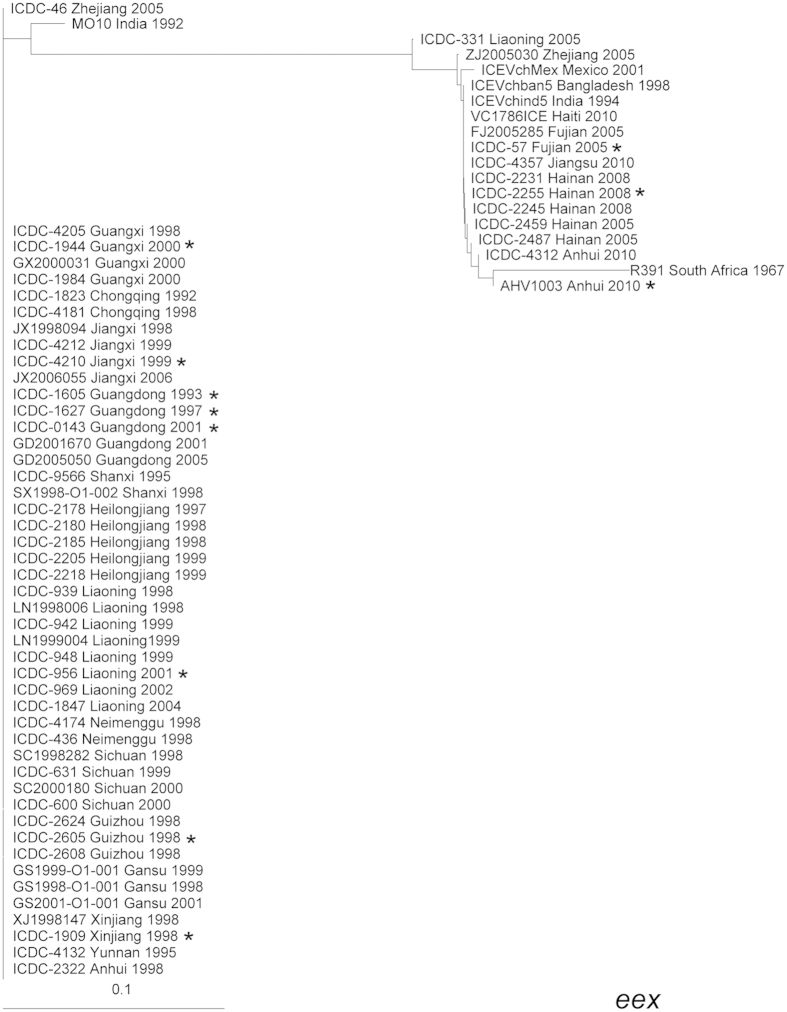
Clustering tree drawn based on the *eex* gene including the 59 *V. cholerae* strains in this study and six representative reference SXT sequences. The whole 432-bp CDS region of the *eex* gene was analyzed. The 11 genomic SXT elements are marked with asterisks.

**Figure 3:**
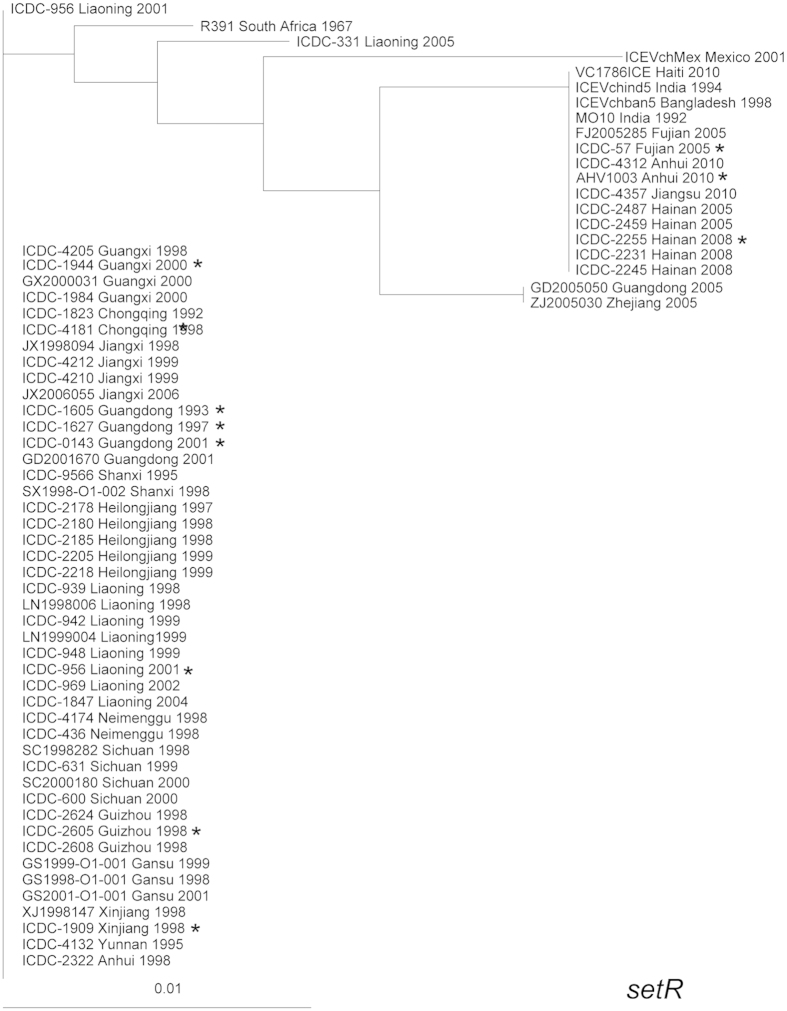
Clustering tree drawn based on the *setR* gene including the 59 *V. cholerae* strains in this study and six representative reference SXT sequences. The whole 648-bp CDS region of the *setR* gene was analyzed. The 11 genomic SXT elements are marked with asterisks.

SetR and SetC/D, the backbone genes located at the extreme 3′- and 5′-ends of the SXT elements, respectively, are the key regulators of SXT elements. Eex and TraG are inner membrane proteins of the donor and recipient cells that mediate entry exclusion in the SXT/R391 family of ICEs and are essential for ICE transfer. Based on the *eex* gene, EexS (SXT) and EexR (R391), the only two exclusion groups in the large family of ICEs, are divided^11^. As shown in Figs 2 and 3, in the cluster trees for the *setR* and *eex* genes, the SXT elements of the Chinese strains were distinctively divided into two groups. Generally, the SXT elements of strains isolated before 2005 were clustered together, while the strains isolated between 2005 and 2010 clustered together in another group. The *eex* and *setR* genes of the strains in the 2005–2010 cluster showed high homology to many SXT elements isolated from other parts of the world, such as the ICE*Vch*Mex1 (Mexico, 2001), ICE*Vch*ind5 (India, 1994), and VC1786ICE (Haiti, 2010) elements. The *eex* genes of the strains in the pre-2005 cluster showed 100% homology only with ICE*Pmi*Usa1 (*Proteus mirabilis* strain HI4320, United States of America, 1986) and ICE*Vch*CHN1307 (*V. cholerae*, China, 1998), which was also identified by our laboratory. Therefore, there was an obvious type substitution for the SXT element in China, although the strains isolated in a region for several consecutive years had SXT elements with essentially the same *eex* and *setR* genes.

### Genetic structure of the SXT elements

Within the 11 strains that have been completely sequenced, the *attL* and *attR* genes are located on same scaffold, and the spaces between these genes are less than 120 Kb, which is similar to the length of the SXT element. BLAST searches with the *prfC* gene showed that the *prfC* genes are adjacent to the *attR* site and have dozens of bases missing from the 5′-end, consistent with the SXT recombination process. The results of these two methods were completely consistent. The 11 SXT elements were designated according to the nomenclature proposed for this family of elements (e.g., ICE*Vch*CHN143)^4^. These elements are listed in Table 1.

The genomes of the 11 detected SXT elements were 9.25–11.03 Kb in length and had 87–102 predicted ORFs (Supplemental Table S1). The sequences of ICE*Vch*CHN2605 and ICE*Vch*CHN143 were found to be similar, except that ICE*Vch*CHN2605 lacked a T at site 97,900. Furthermore, ICE*Vch*CHN1605 was identical to ICE*Vch*CHN143, but was missing 39 bp from positions 97,901 to 97,939. These differences were not related to the coding region; therefore, we grouped these three SXT elements as one subset of ICE.

**Figure 4:**
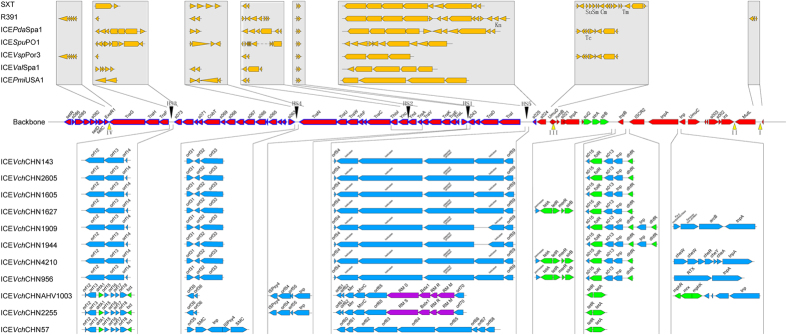
Construction of the 11 SXT elements identified in this study. The 70 ORFs commonly shared by these SXT elements are marked on the backbone, and the ORFs with high homology to *Proteus mirabilis* HI4320 are highlighted in blue. Insertion sequences are listed under the backbone according to their insertion points. The antibiotic resistance genes are highlighted in green, and the RM system genes are marked in purple. The insertions in the five hotspots and four variable regions of seven previously reported ICEs are listed above the backbone as references.

The genetic organization of these 11 SXT ICEs was similar to that of other members of this family (Fig. 4). A total of 70 ORFs were commonly shared by these SXT elements, most of which were included in the set of previously reported conserved core genes^2^. However, an entire 17.8-kb region from *s026* to *s040* was missing from all 11 SXTs in our study, which was different from the ORFs of previously reported ICEs. The ORFs *s084* and *s083* integrated into ORF4 through point deletion of a T at site 267 and a frameshift mutation in *s084*. These replacements may not have an obvious effect on the SXT because deletions of core genes of unknown function, namely, *s082*, *s083*, and *s084*, did not influence SXT transfer frequency. Furthermore, the gene cluster from *s027* to *s040* is not required for SXT^MO10^ mobility or maintenance^8^.

Five conserved insertion hotspots are located between *s043* and *traL* (H1), *traA* and *s054* (H2), *s073* and *traF* (H3), *traN* and *s063* (H4), and *s025* to *traID* (H5)^8^. In H1, only AHV1003 and -2255 have insertions of a cluster of four genes. In H2, all 11 SXT elements have insertions of the same two genes, *s053* (Ync) and *s052* (Ynd). Similarly, all 11 SXT elements have insertions in H3 and H4. Eight SXT elements, namely, ICE*Vch*CHN143, -2605, -1605, -1627, -1909, -1944, -4210, and -956, have the same insertion at these two sites. Among these hotspots, H5 has the largest number of inserted genes and gene combinations. This spot had insertions of four types of gene clusters containing 6–10 ORFs oriented in the same direction as *s025* and *traI*. None of the 11 SXT elements had any insertions in the four variable regions VRI–VRIV. Additionally, the main insertion site of the 11 SXT elements was between *umuc* and *s021*. Three insertion regions were formed at this spot in transposon-like structures, and these regions carried many antibiotic resistance genes. One of these regions was related to the ISCR2 element, which is an IS91-like transposable element that tends to accumulate antibiotic resistance genes.

The restriction-modification system (RM) is a system of genes encoding the functions of DNA modification, recombination, and repair^12^. RM is composed of three polypeptides: R (restriction endonuclease), which recognizes and cut specific DNA sequences; M (modification), which methylates the same sequence to inhibit DNA cleavage and protect the host cell against invasion of foreign DNA; and S (specificity), which determines the specificity of both R and M. In the ICE family, ICE*Vch*Mex1^13^ and ICE*Spu*PO1^14^ encode putative type I RM systems, and ICE*Vsp*Por3 and ICE*Val*Spa1 harbor a putative type III RM system^15^. Two ICEs in our study, AHV1003 and -2255, were found to have a completely similar type I RM system in HS5. This RM was identical to the RM of the *V. cholerae* O1 strain EM-1536 and the *Alteromonas macleodii* strain MED64. However, this RM had very low similarity to the type I RM of ICE*Vch*Mex1, with 18% protein sequence similarity in the R subunit and 14% similarity in the M subunit.

Of the 70 commonly shared ORFs of the 11 SXT elements, 1–49 showed nearly 100% homology to the ORFs between PMI2423 and PMI2482 of *P. mirabilis* strain HI4320 (GenBank: AM942759), which carries a conjugative transposon (PMI2423–PMI2491) similar to the ICE R391 from *P. rettgeri*^16^. The homologies of these ORFs were even higher than their homologies to the ICEs of *V. cholerae* (Supplemental Table S1). The remaining 20 ORFs shared 100% homology to nucleotide sequences of the ICEs of *V. cholerae*, half of which showed no homology to the *P. mirabilis* strain HI4320 genome. This may support the recombination of the different origins of these Chinese SXT ICEs.

Based on the insertion sites of the 11 SXT elements (Fig. 4), we could easily group these elements into two distinct types. The first type included ICE*Vch*CHN2255, ICE*Vch*CHN57, and AHV1003. ICE*Vch*CHN 2255 was more similar to AHV1003 than to ICE*Vch*CHN57. The remaining SXT elements belonged to the second type, which could be further divided into three subsets: the first subset consisted of ICE*Vch*CHN143, -1605, and -2605; the second subset consisted of ICE*Vch*CHN1627, -4210, and -956; and the third subset consisted of ICE*Vch*CHN1909 and -1944. This was consistent with the groups divided based on the *eex* and *setR* genes.

In addition, some backbone genes differed between these two types. For example, the length of the hypothetical protein next to TraL had a difference of six nucleotides between the two types. ORF29, which is adjacent to *s063*, was not present in ICE*Vch*CHN2255, ICE*Vch*CHN57, and AHV1003 because of a ‘T’ to ‘A’ transition at nucleotide 12 and premature termination. *ideA*, i.e., the backbone gene *s062*, is an ICE-encoded DNase that was recently shown to be necessary for inhibiting the natural transformation of Haiti outbreak strains^17^. This gene is 684 bp long and encodes a putative 227-aa-long protein in VC1786ICE. The *ideA* gene of ICE*Vch*CHN57 is 100% homologous to VC1786ICE, but is absent in ICE*Vch*CHN2255 and AHV1003. For the other nine SXT elements, the genes are 675 bp in size, with 90% nucleotide and 91% amino acid similarities to the corresponding sequences in VC1786ICE and ICE*Vch*CHN57.

Detailed information regarding the 11 SXT element sequences reconfirmed that there were two SXT types in the Chinese epidemic O1 *V. cholerae* strains. The major differences between them indicated that they may have different origins and would have evolved independently.

### Antibiotic resistance genes in the SXT elements

The most important feature of SXT elements is that they carry multidrug resistant genes and transfer them between bacteria. Most ICE antibiotic resistance genes are embedded in a composite transposon-like element that interrupts the SXT-encoded *rumAB* operon. As shown in Fig. 4, the antibiotic resistance genes of the SXT elements in this study are also focused in the same region, which is partitioned into the three insertions by transposase. These antibiotic resistance genes appeared to have been obtained through repeated transposition. The genes *sulI*, encoding dihydropteroate synthase and sulfamethoxazole resistance (Su), and *strAB*, encoding streptomycin resistance (Sm), were present in all of the SXT elements analyzed in this study.

In the first insertion fragment, the SXTs of strains 1627, 4210, and 956 contained a resistance gene cluster containing *tetAR*, *merR*, and *strB*; this cluster conferred resistance to tetracycline, mercury, and Su. Insertion 2 contained the largest resistance genes, including the *floR* gene, which confers resistance to chloramphenicol, and the *dhfR* gene, encoding a trimethoprim-resistant dihydrofolate reductase, and was present in eight of the 11 SXT elements (absent in ICE*Vch*CHN2255, ICE*Vch*CHN57, and AHV1003). Furthermore, ICE*Vch*CHN1909 and ICE*Vch*CHN1944 possessed identical copies of the *dhfR* gene separated by the transposase family gene *tnp*. The deduced amino acid sequences of these *dhfR* genes had 69% identity with the *folA* gene of *Riemerella anatipestifer* RA-CH-2, encoding dihydrofolate reductase, and 16% identity with *drf18* of the O139 serogroup strain MO10. Although ICE*Vch*CHN2255, ICE*Vch*CHN57, and AHV1003 lacked the *dhfR* gene at this position, they contained another copy of dihydrofolate reductase, which was identical to the *dfrA1* gene in the class I integron of *Klebsiella pneumoniae* subsp. *pneumoniae* strain B-1104. The *dfrA1* gene was located at an insertion of 5.022 kbp between *traF* and an ORF of unknown function, *orf73*, similar to the position of *dfrA1* from *V. cholerae* O1 serogroup strain C10488^18^. Therefore, in the SXT elements of Chinese El Tor strains, resistance to trimethoprim was encoded either outside or inside the clusters of other resistance genes, but the homology between these two types of trimethoprim resistance determinants was only 27%, and both determinants could not simultaneously exist in one SXT element. In insertion 2, ICE*Vch*CHN2255, ICE*Vch*CHN57, and AHV1003 also contained a copy of the *tetAR* gene. This gene, which encodes a tetracycline efflux protein of class A, was 100% homologous to the ICE*Vsp*Spa1 genes of *V. splendidus*. The *tetAR* gene in insertion 1 belonged to class B and was similar to the genes of *V. cholerae* CP1042. However, there was only 50% homology between these two types of tetracycline-resistance genes. In insertion 3, only ICE*Vch*CHNAHV1003 carried three resistance-related genes, namely, *mphR*, *mrx*, and *mphK*, which confer resistance to erythromycin.

In our previous study profiling the antibiotic resistance of Chinese O1 El Tor strains^1^, the presence of the SXT element was highly correlated with resistance to nalidixic acid, tetracycline, and trimethoprim-sulfamethoxazole. The present study confirmed that the SXT element was indeed a vector of genes conferring resistance to tetracycline and trimethoprim-sulfamethoxazole and genes causing multidrug resistance.

In conclusion, our findings showed that the SXT elements in Chinese epidemic O1 *V. cholerae* strains were split into two types. All of these elements had their own molecular characteristics distinguishing them from the other ICEs and exhibited obvious differences in terms of the sequences of many conserved backbone genes as well as the overall structure. However, strains isolated from a region for several consecutive years were found to have the same SXT type. In 2005, there was an obvious type substitution for the SXT element in the Chinese epidemic strains. Regardless of these differences, SXT elements of both types carried at least five drug resistance genes and played a key role in the emergence of multidrug resistance in O1 serogroup strains.

## Materials and Methods

### Strains

Based on our previous study^1^, 59 strains were retained from the 169 SXT element-positive strains by removal of strains having the same isolation time, region, and antibiotic resistance spectrum. These 59 *V. cholerae* O1 El Tor strains were isolated between 1992 and 2010 from 18 provinces in China. All strains were positive for the *eex*, *setR*, and *int* genes of the SXT element, and 11 of the strains were subjected to whole genome sequencing in our laboratory.

### Polymerase chain reaction (PCR) and sequence alignment

Primer sets were designed for the *eex* (eex-U, 5′-GCT GAT GCA TGA TTT GAT TG-3′; and eex-L, 5′-CAG GCA TCA GGA AGG AAC TG-3′), *setR* (setR-U, 5′-CAC TTC CAT ACC GTC TCC TG-3′; setR-L, 5′-ATC GTT GCT TCT TCA GCT CA-3′), and *int* (int-U, 5′-ATG AGT ACA GCG CCA GAA CC-3′; and int-L, 5′-CGA GCC AAA TGC ACT ACT TG-3′) genes. The PCR products were purified and sequenced. Sequence alignments and comparisons were performed using the Clustal X program (version 2.0), and neighbor joining (NJ) trees were drawn. The random number generator seed was 111, with 1000 bootstrap trials.

### Extraction of SXT element sequences

Raw reads data of the resequenced genomes database of the Chinese O1 El Tor *V. cholerae* set in our laboratory were assembled *de novo* with SOAP*denovo* (V 2.04) using optimal Kmer and minimal coverage parameters. The SXT element sequences were then extracted from the whole genome by two methods. The first was based on the consensus sequence of *attL* and *attR*. According to the alignment of the nucleotide sequences of *V. cholerae* chromosomes on both sides of the SXT element^5^, the consensus sequence could be determined as ‘O’ = ATCATCTCGCACCCTGA. Scaffolds with *attL* and *attR* with a space of less than 120 kb were selected, and the sequences between the two consensus sequences were extracted as the SXT element. The other method was BLAST with the *prfC* gene sequence. Sequences with *attR* following insertion at the 5′-end of the *prfC* gene were extracted as the SXT element.

### Annotation of the SXT elements

Glimmer 3.02 and Rast online prediction tools were used to predict the ORFs and for annotations. The SXT element AY055428 was used as the reference sequence. The genomic sequences of the nine SXT elements have been deposited in GenBank (accession numbers KT151654 through KT151664).

## Additional Information

**How to cite this article**: Wang, R. *et al.* Variations in SXT elements in epidemic *Vibrio cholerae* O1 El Tor strains in China. *Sci. Rep.* **6**, 22733; doi: 10.1038/srep22733 (2016).

## Supplementary Material

Supplementary Information

## Figures and Tables

**Table 1:** Strains carrying the SXT element and the molecular characteristics of their SXT elements.

Strain	Province	Source	Year isolated	Length of SXT (bp)	Number of ORFs	G+C (%)	Resistance genes
ICDC-VC1605	Guangdong	Patient	1993	98419	87	47.43	*sul2*, *strAB*, *dhfR*, *folR*
ICDC-VC1627	Guangdong	Patient	1997	102413	92	47.93	*sul2*, *strAB*, *dhfR*, *tetAR*, *merR*, *folR*
ICDC-VC2605	Guizhou	Patient	1998	98458	87	47.42	*sul2*, *strAB*, *dhfR*, *folR*
ICDC-VC1909	Xinjiang	Patient	1998	108995	93	47.47	*sul2*, *strAB*, *dhfR*, *folR*
ICDC-VC4210	Jiangxi	Patient	1999	110349	99	47.81	*sul2*, *strAB*, *dhfR*, *tetAR*, *merR*, *folR*
ICDC-VC1944	Guangxi	Unknown	2000	100703	89	47.45	*sul2*, *strAB*, *dhfR*, *folR*
ICDC-VC0143	Guangdong	Patient	2001	98459	87	47.42	*sul2*, *strAB*, *dhfR*, *folR*
ICDC-VC0956	Liaoning	Water	2001	109322	94	47.89	*sul2*, *strAB*, *dhfR*, *tetAR*, *merR*, *folR*
ICDC-VC57	Fujian	Patient	2005	96153	93	46.24	*dfrA*1, *bcr*, *sul2*, *strAB*, *tetAR,*
ICDC-VC2255	Hainan	Patient	2008	92592	95	46.51	*dfrA*1, *bcr*, *sul2*, *strAB*, *tetAR*
AHV1003	Anhui	Patient	2010	101856	102	47.46	*dfrA*1, *bcr*, *sul2*, *strAB*, *tetAR*, *mphRK*, *mrx*
