# Supplementary material for: Variations in SXT elements in epidemic Vibrio cholerae O1 El Tor strains in China
Source: Sci Rep. 2016 Mar 9;6:22733. doi: 10.1038/srep22733 (PMC4783696; doi:10.1038/srep22733)
Supplement: Supplementary Information [file srep22733-s1.pdf]

Supplementary Information:

Variations in SXT elements in epidemic *Vibrio cholerae* O1 El Tor strains in China

Ruibai Wang[1,3], Dong Yu[2] , Junjie Yue[2*], Biao Kan[1,3*]

Supplement table 1. The predicted ORFs of every SXT genome and their identities

57		AHV1003		2255		1944		1909		956		4210		1627		143		1605		2605		Identities(%)	
ORF	Description	ORF	Description	ORF	Description	ORF	Description	ORF	Description	ORF	Description	ORF	Description	ORF	Description	ORF	Description	ORF	Description	ORF	Description	*P. mirabilis* strain HI4320	ICEs of *V. cholerae*
1	setR	1	setR	1	setR	1	setR	1	setR	1	setR	1	setR	1	setR	1	setR	1	setR	1	setR	100	98
2	s086	2	s086	2	s086	2	s086	2	s086	2	s086	2	s086	2	s086	2	s086	2	s086	2	s086	100	99
3	s085	3	s085	3	s085	3	s085	3	s085	3	s085	3	s085	3	s085	3	s085	3	s085	3	s085	100	99
4	hypothetical	4	hypothetical	4	hypothetical	4	hypothetical	4	hypothetical	4	hypothetical	4	hypothetical	4	hypothetical	4	hypothetical	4	hypothetical	4	hypothetical	100	99
5	s082	5	s082	5	s082	5	s082	5	s082	5	s082	5	s082	5	s082	5	s082	5	s082	5	s082	100	97
6	setD	6	setD	6	setD	6	setD	6	setD	6	setD	6	setD	6	setD	6	setD	6	setD	6	setD	100	97
7	setC	7	setC	7	setC	7	setC	7	setC	7	setC	7	setC	7	setC	7	setC	7	setC	7	setC	99	97
8	EexR1	8	EexR1	8	EexR1	8	EexR1	8	EexR1	8	EexR1	8	EexR1	8	EexR1	8	EexR1	8	EexR1	8	EexR1	100	97
9	TraG	9	TraG	9	TraG	9	TraG	9	TraG	9	TraG	9	TraG	9	TraG	9	TraG	9	TraG	9	TraG	99	96
10	TraH	10	TraH	10	TraH	10	TraH	10	TraH	10	TraH	10	TraH	10	TraH	10	TraH	10	TraH	10	TraH	100	97
11	TraF	11	TraF	11	TraF	11	TraF	11	TraF	11	TraF	11	TraF	11	TraF	11	TraF	11	TraF	11	TraF	100	95
12	hypothetical	12	hypothetical	12	hypothetical	12	pcrA	12	pcrA	12	pcrA	12	pcrA	12	pcrA	12	pcrA	12	pcrA	12	pcrA		
13	IntIPac	13	IntIPac	13	IntIPac	13	endonuclease	13	endonuclease	13	endonuclease	13	endonuclease	13	endonuclease	13	endonuclease	13	endonuclease	13	endonuclease		
14	drfA1	14	drfA1	14	drfA1	14	hypothetical	14	hypothetical	14	hypothetical	14	hypothetical	14	hypothetical	14	hypothetical	14	hypothetical	14	hypothetical		
15	hypothetical	15	hypothetical	15	hypothetical																		
16	Membrane	16	membrane	16	membrane																		
17	hypothetical	17	hypothetical	17	hypothetical																		
18	hypothetical	18	hypothetical	18	hypothetical																		
19	bct	19	bct	19	bct																		
20	s073	20	s073	20	s073	15	s073	15	s073	15	s073	15	s073	15	s073	15	s073	15	s073	15	s073	100	93
21	s072	21	s072	21	phage	16	phage	16	phage	16	phage	16	phage	16	phage	16	phage	16	phage	16	phage	100	98
22	hypothetical	22	hypothetical	22	hypothetical	17	hypothetical	17	hypothetical	17	hypothetical	17	hypothetical	17	hypothetical	17	hypothetical	17	hypothetical	17	hypothetical	100	98
23	s071	23	s071	23	s071	18	s071	18	s071	18	s071	18	s071	18	s071	18	s071	18	s071	18	s071	100	98
24	Plasmid	24	CobT	24	CobT	19	CobT	19	CobT	19	CobT	19	CobT	19	CobT	19	CobT	19	CobT	19	CobT	99	98
25	s069	25	s069	25	s069	20	s069	20	s069	20	s069	20	s069	20	s069	20	s069	20	s069	20	s069	100	99
26	s068	26	s068	26	s068	21	s068	21	s068	21	s068	21	s068	21	s068	21	s068	21	s068	21	s068	100	98
27	hypothetical	27	hypothetical	27	hypothetical	22	hypothetical	22	hypothetical	22	hypothetical	22	hypothetical	22	hypothetical	22	hypothetical	22	hypothetical	22	hypothetical	99	98
28	CobS	28	CobS	28	s067	23	s067	23	s067	23	s067	23	s067	23	s067	23	s067	23	s067	23	s067	99	98
29	s066	29	s066	29	s066	24	s066	24	s066	24	s066	24	s066	24	s066	24	s066	24	s066	24	s066	100	97
30	hypothetical	30	hypothetical	30	hypothetical	25	hypothetical	25	hypothetical	25	hypothetical	25	hypothetical	25	hypothetical	25	hypothetical	25	hypothetical	25	hypothetical	100	100
31	s065	31	s065	31	s065	26	s065	26	s065	26	s065	26	s065	26	s065	26	s065	26	s065	26	s065	100	99
32	ssd	32	ssd	32	ssd	27	ssd	27	ssd	27	ssd	27	ssd	27	ssd	27	ssd	27	ssd	27	ssd	100	98
33	hypothetical	33	hypothetical	33	hypothetical	28	hypothetical	28	hypothetical	28	hypothetical	28	hypothetical	28	hypothetical	28	hypothetical	28	hypothetical	28	hypothetical	100	98
						29	hypothetical	29	hypothetical	29	hypothetical	29	hypothetical	29	hypothetical	29	hypothetical	29	hypothetical	29	hypothetical	100	99
34	s063	34	s063	34	s063	30	s063	30	s063	30	s063	30	s063	30	s063	30	s063	30	s063	30	s063	100	97
35	Dns	35	hypothetical	35	hypothetical	31	Dns	31	Dns	31	Dns	31	Dns	31	Dns	31	Dns	31	Dns	31	Dns		
36	SMC	36	hypothetical	36	hypothetical	32	regulatory	32	regulatory	32	regulatory	32	regulatory	32	regulatory	32	regulatory	32	regulatory	32	regulatory		
37	transposase					33	membrane	33	membrane	33	membrane	33	membrane	33	membrane	33	membrane	33	membrane	33	membrane		
38	ISPsy4																						
39	SMC																						
40	TraN	37	TraN	37	TraN	34	TraN	34	TraN	34	TraN	34	TraN	34	TraN	34	TraN	34	TraN	34	TraN	99	96
41	TraU	38	TraU	38	TraU	35	TraU	35	TraU	35	TraU	35	TraU	35	TraU	35	TraU	35	TraU	35	TraU	100	98
42	TraW	39	TraW	39	TraW	36	TraW	36	TraW	36	TraW	36	TraW	36	TraW	36	TraW	36	TraW	36	TraW	100	98

57		AHV1003		2255		1944		1909		956		4210		1627		143		1605		2605		Identities(%)	
ORF	Description	ORF	Description	ORF	Description	ORF	Description	ORF	Description	ORF	Description	ORF	Description	ORF	Description	ORF	Description	ORF	Description	ORF	Description	*P. mirabilis* strain HI4320	ICEs of *V. cholerae*
43	TrhF	40	TrhF	40	TrhF	37	TrhF	37	TrhF	37	TrhF	37	TrhF	37	TrhF	37	TrhF	37	TrhF	37	TrhF	99	97
44	345	41	hypothetical	41	hypothetical	38	hypothetical	38	hypothetical	38	hypothetical	38	hypothetical	38	hypothetical	38	hypothetical	38	hypothetical	38	hypothetical	100	98
45	TraC	42	TraC	42	TraC	39	TraC	39	TraC	39	TraC	39	TraC	39	TraC	39	TraC	39	TraC	39	TraC	100	97
46	s054	43	Thiol	43	Thiol	40	Thiol	40	Thiol	40	Thiol	40	Thiol	40	Thiol	40	Thiol	40	Thiol	40	Thiol	99	98
47	s053(Ync)	44	Ync	44	Ync	41	Ync	41	Ync	41	Ync	41	Ync	41	Ync	41	Ync	41	Ync	41	Ync	100	99
48	s052(Ynd)	45	Ynd	45	Ynd	42	Ynd	42	Ynd	42	Ynd	42	Ynd	42	Ynd	42	Ynd	42	Ynd	42	Ynd	100	100
49	TraA	46	TraA	46	TraA	43	TraA	43	TraA	43	TraA	43	TraA	43	TraA	43	TraA	43	TraA	43	TraA	100	98
50	TraV	47	TraV	47	traV	44	traV	44	traV	44	traV	44	traV	44	traV	44	traV	44	traV	44	traV	100	99
51	TraB	48	TraB	48	TraB	45	TraB	45	TraB	45	TraB	45	TraB	45	TraB	45	TraB	45	TraB	45	TraB	100	98
52	TraK	49	TraK	49	TraK	46	TraK	46	TraK	46	TraK	46	TraK	46	TraK	46	TraK	46	TraK	46	TraK	100	97
53	TraE	50	TraE	50	TraE	47	TraE	47	TraE	47	TraE	47	TraE	47	TraE	47	TraE	47	TraE	47	TraE	100	99
54	TraL	51	TraL	51	TraL	48	TraL	48	TraL	48	TraL	48	TraL	48	TraL	48	TraL	48	TraL	48	TraL	100	99
55	hypothetical	52	hypothetical	52	hypothetical	49	hypothetical	49	hypothetical	49	hypothetical	49	hypothetical	49	hypothetical	49	hypothetical	49	hypothetical	49	hypothetical	100	100
		53	ISPsy4	53	ISPsy4																		
		54	hypothetical	54	hypothetical																		
		55	hypothetical	55	hypothetical																		
		56	Transposase	56	Transposase																		
56	s043	57	s043	57	s043	50	s043	50	s043	50	s043	50	s043	50	s043	50	s043	50	s043	50	s043	100	96
57	234	58	234	58	234	51	234	51	234	51	234	51	234	51	234	51	234	51	234	51	234	100	98
58	TraD	59	TraD	59	TraD	52	TraD	52	TraD	52	TraD	52	TraD	52	TraD	52	TraD	52	TraD	52	TraD	100	96
59	TraI	60	TraI	60	TraI	53	TraI	53	TraI	53	TraI	53	TraI	53	TraI	53	TraI	53	TraI	53	TraI	100	95
60	hypothetical	61	hypothetical	61	hypothetical	54	hypothetical	54	hypothetical	54	hypothetical	54	hypothetical	54	hypothetical	54	hypothetical	54	hypothetical	54	hypothetical		
61	hypothetical	62	hypothetical	62	hypothetical	55	helicase	55	helicase	55	helicase	55	helicase	55	helicase	55	helicase	55	helicase	55	helicase		
62	protease	63	Mrr	63	Mrr	56	Helicase	56	Helicase	56	Helicase	56	Helicase	56	Helicase	56	Helicase	56	Helicase	56	Helicase		
63	hypothetical	64	McrC	64	hypothetical	57	plasmid	57	plasmid	57	plasmid	57	plasmid	57	plasmid	57	plasmid	57	plasmid	57	plasmid		
64	Type II RM	65	enzyme	65	ATPase	58	helicase	58	helicase	58	helicase	58	helicase	58	helicase	58	helicase	58	helicase	58	helicase		
65	hypothetical	66	Type I RM subunit R	66	Type I RM subunit R	59	hypothetical	59	hypothetical	59	hypothetical	59	hypothetical	59	hypothetical	59	hypothetical	59	hypothetical	59	hypothetical		
66	hypothetical	67	bstXI	67	bstXI																		
67	hypothetical	68	Type I RM subunit S	68	Type I RM subunit S																		
68	COGs COG2378	69	Type I RM subunit M	69	Type I RM subunit M																		
		70	COGs COG2378	70	hypothetical																		
69	hypothetical	71	hypothetical	71	hypothetical	60	hypothetical	60	hypothetical	60	hypothetical	60	hypothetical	60	hypothetical	60	hypothetical	60	hypothetical	60	hypothetical	100	96
70	polymerase III	72	polymerase III	72	polymerase III	61	polymerase III	61	polymerase III	61	polymerase III	61	polymerase III	61	polymerase III	61	polymerase III	61	polymerase III	61	polymerase III	97	99
71	hypothetical	73	hypothetical	73	hypothetical	62	hypothetical	62	hypothetical	62	hypothetical	62	hypothetical	62	hypothetical	62	hypothetical	62	hypothetical	62	hypothetical	96	100
72	UmuD	74	UmuD	74	UmuD	63	UmuD	63	UmuD	63	UmuD	63	UmuD	63	UmuD	63	UmuD	63	UmuD	63	UmuD	97	100
73	'rumB	75	'rumB	75	rumB	64	rumB	64	rumB	64	rumB	64	rumB	64	rumB	64	rumB	64	rumB	64	rumB	97	100
74	s021	76	s021	76	s021	65	s021	65	s021	65	s021	65	s021	65	s021	65	s021	65	s021	65	s021	—	100
75	tnpA	77	tnpA'	77	tnpA	66	tnpA	66	tnpA	66	tnpA	66	tnpA	66	tnpA	66	tnpA	66	tnpA	66	tnpA	—	100
76	hypothetical	78	hypothetical	78	hypothetical	67	hypothetical	67	hypothetical	67	hypothetical	67	hypothetical	67	hypothetical	67	hypothetical	67	hypothetical	67	hypothetical	—	100
77	sul2	79	sul2	79	sul2	68	sul2	68	sul2	68	sul2	68	sul2	68	sul2	68	sul2	68	sul2	68	sul2	—	100
78	strA	80	strA	80	strA	69	strA	69	strA	69	strA	69	strA	69	strA	69	strA	69	strA	69	strA	—	100

57		AHV1003		2255		1944		1909		956		4210		1627		143		1605		2605		Identities(%)	
ORF	Description	ORF	Description	ORF	Description	ORF	Description	ORF	Description	ORF	Description	ORF	Description	ORF	Description	ORF	Description	ORF	Description	ORF	Description	*P. mirabilis* strain HI4320	ICEs of *V. cholerae*
79	strB	81	strB	81	strB	70	strB	70	strB	70	strB	70	strB	70	strB	70	strB	70	strB	70	strB	—	100
										71	Permease	71	Permease	71	Permease								
										72	tetA	72	tetA	72	tetA								
										73	tetR	73	tetR	73	tetR								
										74	MerR	74	MerR	74	MerR								
										75	StrB	75	StrB	75	StrB								
80	tnpB	82	tnpB	82	tnpB	71	tnpB	71	tnpB	76	tnpB	76	tnpB	76	tnpB	71	tnpB	71	tnpB	71	tnpB	—	100
81	tetR	83	tetR	83	tetR	72	s015	72	s015	77	s015	77	s015	77	s015	72	s015	72	s015	72	s015		
82	tetA	84	tetA	84	tetA	73	folR	73	folR	78	folR	78	folR	78	folR	73	folR	73	folR	73	folR		
						74	s013	74	s013	79	s013	79	s013	79	s013	74	s013	74	s013	74	s013		
						75	transposase	75	transposase	80	transposase	80	transposase	80	transposase	75	transposase	75	transposase	75	transposase		
						76	dhfR	76	dhfR	81	dhfR	81	dhfR	81	dhfR	76	dhfR	76	dhfR	76	dhfR		
						77	transposase	77	transposase														
						78	dhfR	78	dhfR														
83	tnpB	85	tnpB	85	tnpB	79	tnpB	79	tnpB	82	tnpB	82	tnpB	82	tnpB	77	tnpB	77	tnpB	77	tnpB	—	100
84	tnpA	86	tnpA	86	tnpA	80	tnpA	80	tnpA	83	tnpA	83	tnpA	83	tnpA	78	tnpA	78	tnpA	78	tnpA	—	99
		87	mphR					81	oxidoreductase	84	RTX	84	CheW										
		88	mrx					82	Sensory	85	tnpA	85	CheB										
		89	mphK					83	arcB			86	heatrepeat										
		90	hypothetical					84	tnpA			87	CheR										
		91	hypothetical									88	CheY										
		92	hypothetical									89	CheA										
		93	transposase									90	tnpA										
85	tnp	94	tnp	87	tnp	81	tnp	85	tnp	86	tnp	91	tnp	84	tnp	79	tnp	79	tnp	79	tnp	—	100
86	UmuC	95	UmuC	88	UmuC	82	UmuC	86	UmuC	87	UmuC	92	UmuC	85	UmuC	80	UmuC	80	UmuC	80	UmuC	95	100
87	hypothetical	96	hypothetical	89	hypothetical	83	hypothetical	87	hypothetical	88	hypothetical	93	hypothetical	86	hypothetical	81	hypothetical	81	hypothetical	81	hypothetical	95	100
88	hypothetical	97	hypothetical	90	hypothetical	84	hypothetical	88	hypothetical	89	hypothetical	94	hypothetical	87	hypothetical	82	hypothetical	82	hypothetical	82	hypothetical	95	100
89	s003	98	s003	91	s003	85	s003	89	s003	90	s003	95	s003	88	s003	83	s003	83	s003	83	s003	97	100
90	s002	99	s002	92	s002	86	s002	90	s002	91	s002	96	s002	89	s002	84	s002	84	s002	84	s002	97	100
91	integrase	100	integrase	93	integrase	87	integrase	91	integrase	92	integrase	97	integrase	90	integrase	85	integrase	85	integrase	85	integrase	97	100
92	MutL	101	MutL	94	MutL	88	MutL	92	MutL	93	MutL	98	MutL	91	MutL	86	MutL	86	MutL	86	MutL	—	100
93	hypothetical	102	hypothetical	95	hypothetical	89	hypothetical	93	hypothetical	94	hypothetical	99	hypothetical	92	hypothetical	87	hypothetical	87	hypothetical	87	hypothetical	100	100
